# Microstructure and Durability Performance of Mortars with Volcanic Powder from Calbuco Volcano (Chile) after 4 Hardening Years

**DOI:** 10.3390/ma14071751

**Published:** 2021-04-02

**Authors:** Rosa María Tremiño, Teresa Real-Herraiz, Viviana Letelier, José Marcos Ortega

**Affiliations:** 1 Departamento de Ingeniería Civil, Universidad de Alicante, Ap. Correos 99, 03080 Alicante, Spain; rmta2@alu.ua.es; 2 Instituto de Matemática Multidisciplinar, Universidad Politécnica de Valencia, Camino de Vera s/n, 46022 Valencia, Spain; tereaher@upv.es; 3 Departamento de Obras Civiles, Universidad de la Frontera, Av. Fco. Salazar, Temuco 01145, Chile; viviana.letelier@ufrontera.cl

**Keywords:** volcanic powder, Calbuco volcano, very long term, microstructure, durability

## Abstract

One of the most popular ways to lessen the impact of the cement industry on the environment consists of substituting clinker by additions. The service life required for real construction elements is generally long, so it would be interesting to obtain information about the effects of new additions after a hardening period of several years. Analyzed here are the effects of the incorporation of volcanic ashes, coming from Calbuco volcano’s last eruption (Chile), as clinker replacement, in the durability and pore structure of mortars, after approximately 4 hardening years (1500 days), in comparison with reference specimens without additions. The substitution percentages of clinker by volcanic powder studied were 10% and 20%. The microstructure was characterized with mercury intrusion porosimetry and impedance spectroscopy. In order to evaluate the pozzolanic activity of the volcanic powder after 1500 days, differential thermal analyses were performed. Water absorption after immersion, steady-state diffusion coefficient and length change were also studied. In accordance with the results obtained, the 10% and 20% substitution of clinker by volcanic powder from the Calbuco volcano showed beneficial effects in the mortars after 4 years, especially regarding the microstructure and chloride diffusion, without noticeable influence in their water absorption.

## 1. Introduction

At present, the cement industry still constitutes an important pollutant sector [1,2], and it is necessary that this industry contributes to the current worldwide goals regarding global warming reduction. Therefore, for lessening its greenhouse gas emissions and making the cement industry and construction sector in general more sustainable, several strategies have been developed, such as the research on eco-friendly materials, which has experienced great progress in recent years [3,4,5].

With respect to cement-based materials, one of the most popular ways of reducing the greenhouse gas emissions, due to their manufacture, consists of substituting clinker with supplementary cementitious materials [6,7,8,9]. Some of them are residues generated in other industrial processes, so its reuse is also beneficial from an environmental perspective. Among the supplementary cementitious materials available, it is important to highlight silica fume [10,11], ground granulated blast-furnace slag [12,13], and fly ash [14], which have all been commonly used in recent decades. Despite that, developing alternatives to these classical additions is still an important topic of research. Among others, some examples of these new supplementary cementitious materials are brick powder [15], rice husk ash [16], glass powder [17,18], red mud [19], etc.

In relation to the possible use of powders coming from volcanic materials as an addition to cement, research has shown that they would provide adequate service properties [20,21]. On the one hand, it has been reported that these volcanic powders can develop pozzolanic activity [20] and improve the durability of cement-based materials [22,23]. Nevertheless, other works indicated that the incorporation of volcanic powders as a substitution of clinker produced a reduction incompressive strength [24,25] and an increase in porosity [21,26], recommending percentages of this addition up to 20%, inwhich an approximate 23% reduction in strength has been reported [25]. In addition to this, it is important to highlight that the majority of these studies have analyzed the effects of volcanic powder at relatively short hardening ages [27].

On the other hand, the service life required for construction elements, belonging to real structures, buildings, and other engineering works is generally long [28]. Then, to assess if these new additions to cement-based materials, such as volcanic powder, are suitable for use in real construction elements, it would be very interesting to obtain information about their behavior in the very long term, after a hardening period of several years. Furthermore, this could be particularly relevant in the case of pozzolanic materials.

The main purpose of this research is to study the effects of the incorporation of volcanic powder, coming from the last eruption of the Calbuco volcano (Chile), as clinker replacement, in the durability and pore structure of mortars after approximately 4 hardening years (1500 days), compared to reference mortars without additions. The analyzed percentages of clinker substituted by volcanic powder were 10% and 20%. The pore structure of the mortars was characterized using mercury intrusion porosimetry and the non-destructive impedance spectroscopy technique. For evaluating the development of the possible pozzolanic activity of the volcanic powder at the end of the time period studied, differential thermal analyses were performed. With respect to the durability of the mortars, the steady-state diffusion coefficient, determined from the resistivity of the water-saturated samples and the water absorption after immersion, were obtained. Lastly, the length change in the mortars was measured after 1500 hardening days for assessing the possible effects of the volcanic powder addition in this respect.

## 2. Materials and Methods

### 2.1. Materials and Sample Preparation

**Figure 1:**
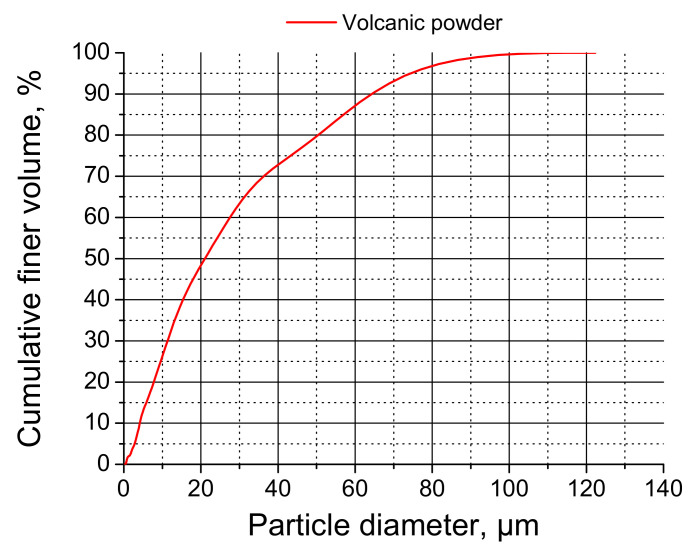
Size distribution of particles of studied powder.

The volcanic powder (VP) studied in this research came from Calbuco volcano’s last eruption (41°20′ S, 72°37′ W, 2003 m.a.s.l.). This volcano is situated in the southern Andes, between the cities of Ensenada and Puerto Montt in Chile. The dates of the last subplinian eruption of this volcano were April 22nd–23rd, 2015. Porphyritic-basaltic andesite (~55% by weight of SiO_2_) [29] was detected in the erupted debris. This fallout mostly affected the volcano’s northeast area, although the finest ashes reached southern Chile and Argentinean Patagonia, where they mainly settled. The distribution of the particle size of the debris showed particle fractions ranging between 3 and 350 µm [30]. In this study, the volcanic powder was sieved, and the particles used as addition were those with sizes smaller than 75 µm. The chemical components of the VP are shown in Table 1 [27], whereas the size distribution of the powder is represented in Figure 1 [27].

Three kinds of mortar were studied in this research, in which Calbuco’s VP and Portland cement were combined. This cement was type CEM I 42.5 R [31]. Firstly, a reference mortar without VP, designed as REF in the results and discussion sections, was prepared. On the other hand, mortars with two different VP binders were also made. These binders incorporated 10% and 20% in weight as cement CEM I 42.5 R substitution, named VP10 and VP20, respectively. A water/cement ratio of 0.5 was used for all of the studied mortars, as well as an aggregate/cement ratio equal to 3. The fine quartz sand accomplished the provisions of standard UNE-EN 196-1 [32] and was provided by the company Normensand (Beckum, Germany) [33,34,35].

Two types of sample were cast. One of them consisted of cylindrical samples with dimensions 10 cm in diameter, and 15 cm in height. In addition, prismatic specimens with the dimensions of 25 mm × 25 mm × 285 mm were also made. All of them were stored in a chamber at 20°C at a 95% relative humidity (RH) during the initial 24 h. After that curing period, they were de-moulded. In order to optimize the total amount of material required and their storage, cylindrical samples were cut to obtain slices with 1 cm in height. Finally, the samples were kept in an optimum laboratory condition (20 °C and 100% RH) until 1500 hardening days (approximately 4 years), when they were tested. This condition consisted of storing the specimens in hermetically sealed containers, which contained distilled water in their bottom part to achieve a 100% relative humidity environment. The mortar samples were placed into the containers, avoiding contact with water by using a rack above the water level. In addition, these containers were kept in a chamber at a controlled temperature of 20 °C. The dimensions of the hermetically sealed containers used were 325 mm × 265 mm × 150 mm.

### 2.2. Mercury Intrusion Porosimetry

Mercury intrusion porosimetry is useful for obtaining data regarding the pore network of materials [36,37,38]. Here, the porosimetry test was performed using a Poremaster-60 GT porosimeter manufactured by Quantachrome Instruments (Boynton Beach, FL, USA). The specimens were oven-dried at 50 °C over 48 h before performing the test. The results analyzed in this research were total porosity, pore size distribution, and percentage of Hg retained at the end of the experiment. The pore size distribution was analyzed, taking into account the next intervals: <10 nm, 10–100 nm, 100 nm to 1μm, 1–10 μm, 10 μm to 0.1 mm, and >0.1 mm [39,40]. Two measurements were made on each type of mortar at the hardening age studied. Pieces taken from the 1 cm disks, with irregular shapes, were tested. The total weight of the pieces tested in each of the measurements was approximately 1.5 g.

### 2.3. Impedance Spectroscopy

The impedance spectroscopy technique is used for obtaining data related to the microstructure of cement-based materials [41,42,43,44]. In this research, the impedance spectroscopy measurements were done with an analyzer model Agilent 4294A (Agilent Technologies, Kobe, Japan). This device allows measurements between 10^−14^ and 0.1 F. The electrodes were circular (diameter 8 cm). They were made with flexible graphite, which was fixed to a copper disk, also measuring 8 cm in diameter. The range of frequencies was 100 Hz to 100 MHz.

**Figure 2:**
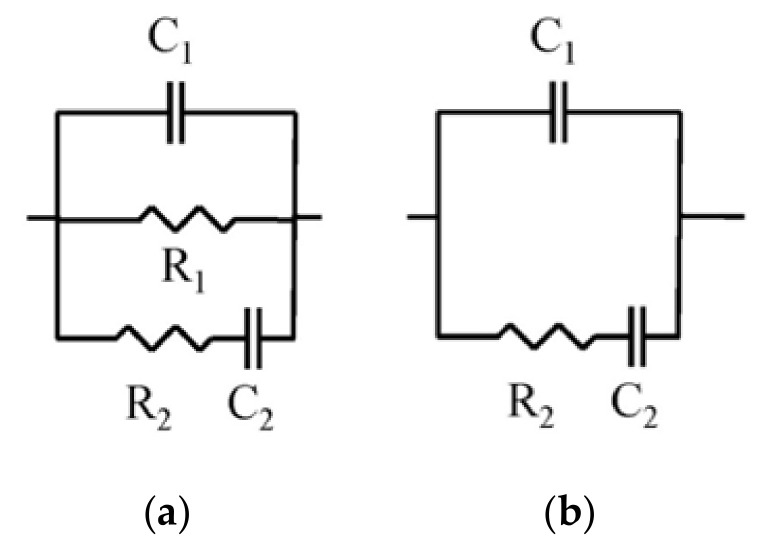
(**a**) Equivalent circuit for the contacting method [18,27]; (**b**) Equivalent circuit for the non-contacting method [18,27]. Reprinted with permission from [18,27] 2018 MDPI.

Contacting and non-contacting measurements were conducted [41]. The experimental records were fit to the equivalent circuits shown in Figure 2a,b [41], which consisted of several resistances and capacitances [41]. The resistance R_1_ provides data about the percolating pores in the material [41], the resistance R_2_ provides information about the overall pores [41], the capacitance C_1_ provides information regarding a sample’s solid fraction [41], and the C_2_ capacitance is associated with the pores’ surface contacting with the electrolyte present inside the pore network [42]. In this work, regarding the pore structure characterization, only the results of the parameters R_2_, C_1_ and C_2_, determined using the non-contacting method have been analyzed, on account of their greater accuracy. The R_1_ resistance, which can be determined using the contacting method, was only used for calculating the steady-state chloride diffusion coefficient in water-saturated specimens, as will be explained in Section 2.6. Eight different slices, measuring 1 cm in height, were analyzed with this technique for each mortar type.

### 2.4. Differential Thermal Analysis

The differential thermal analyses were conducted with a simultaneous TG-DTA model TGA/SDTA851e/SF/1100 from Mettler Toledo (Columbus, OH, USA), which allows operation from room temperature, up to 1100 °C. The heating ramp selected was 20 °C/min up to 1000 °C in N_2_ atmosphere. The curve weight derivate versus temperature was determined. Three measurements were made on each type of mortar at the hardening age studied. The powder samples tested with this technique were obtained from milling pieces taken from disks of 1 cm in thickness. The total weight of powder in each one the measurements was approximately 20 mg.

### 2.5. Water Absorption

The absorption after immersion was determined following the procedure described in the ASTM Standard C642-06 [45]. Six pieces, taken from slices of 1 cm in height, were tested for each kind of mortar studied.

### 2.6. Steady-State Diffusion Coefficient

The steady-state chloride diffusion coefficient was determined from the electrical resistivity of the samples. The resistivity was calculated from the impedance resistance R_1_ measured in water saturated samples. As explained previously, the resistance R_1_ is directly related to the crossing pores of the material [41] and is therefore equivalent to the electrical resistance of the sample [46]. This test has been successfully used in several research works for different types of cement-based materials [46,47]. For each binder, six different samples of 1 cm in thickness were tested. Finally, the steady-state diffusion coefficient was obtained using the following Equation [48]

(1) $$D_{S}=\frac{2\times 10^{-10}}{\rho }$$

where D_s_ is the chloride steady-state diffusion coefficient through the sample (m^2^/s), and ρ is the electrical resistivity of the specimen (Ω·m).

### 2.7. Shrinkage/Expansion

In order to assess if the incorporation of volcanic powder from the Calbuco volcano resulted in the development of the expansion or shrinkage phenomena in the mortars, their length change was measured after1500 hardening days. For each mortar type, six specimens with dimensions measuring 25 mm × 25 mm × 285 mm were tested. The length change in percentage after 1500 hardening days was determined from the measurements performed with a length comparator according to ASTM Standard C596-01 [49].

## 3. Results

### 3.1. Mercury Intrusion Porosimetry

**Figure 3:**
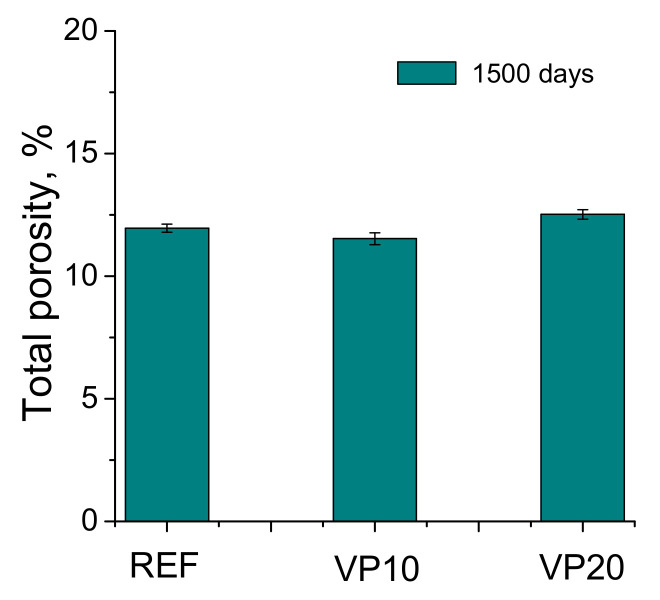
Total porosity results for the analyzed series.

The total porosity results after1500 days are represented in Figure 3. This porosity was slightly greater for the VP20 mortars in comparison to the REF and VP10 ones. Despite that, the total porosity was relatively similar for the different series studied.

**Figure 4:**
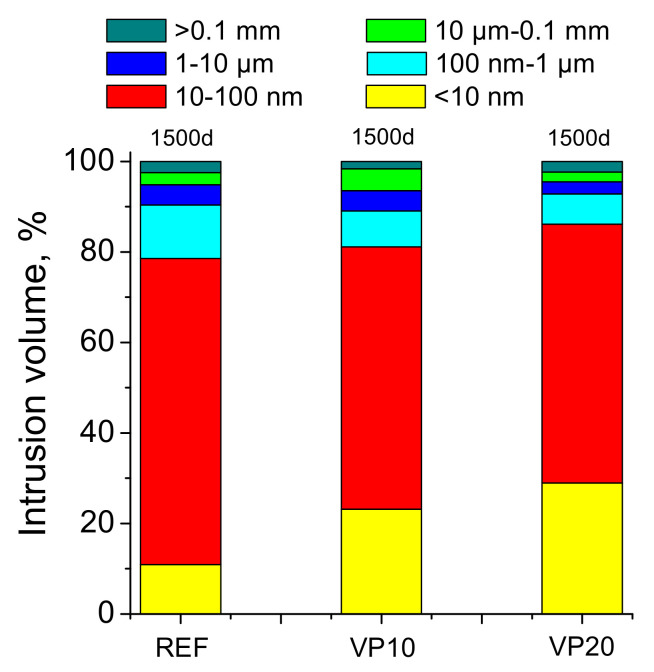
Pore size distributions noted for the series studied.

Regarding the pore size distributions of the analyzed mortar series, they are depicted in Figure 4. The proportion of pores with lower sizes was higher for specimens which incorporated volcanic powder in comparison with REF mortars. In this regard, it is important to highlight the greater percentage of pores with diameters in the range of <10 nm, noted for VP10 and VP20 mortars. In view of these results, the addition of volcanic powder gave a more refined pore network to the mortars after 1500 hardening days.

**Figure 5:**
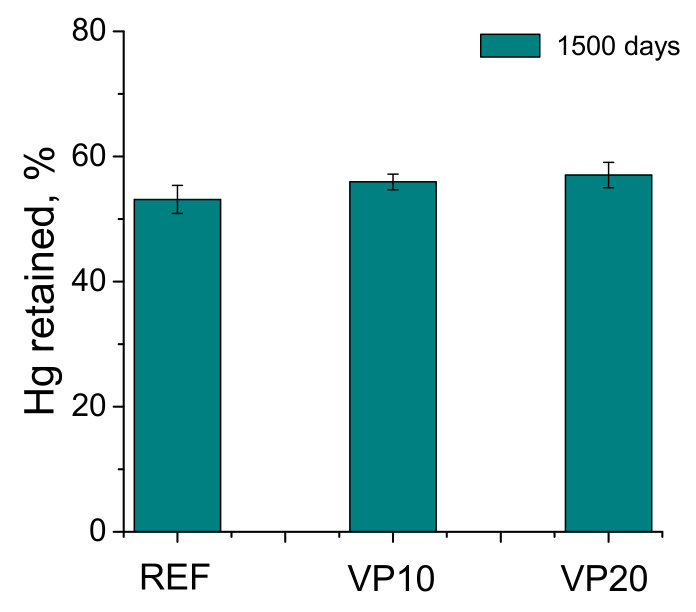
Results of percentage of mercury retained at the end of porosimetry test for the analyzed series.

The results of the percentage of mercury retained at the end of the porosimetry test are depicted in Figure 5. This percentage was greater for specimens with the addition, in comparison to those taken as reference. The highest percentage of mercury retained was noted for VP20 mortars.

### 3.2. Impedance Spectroscopy

**Figure 6:**
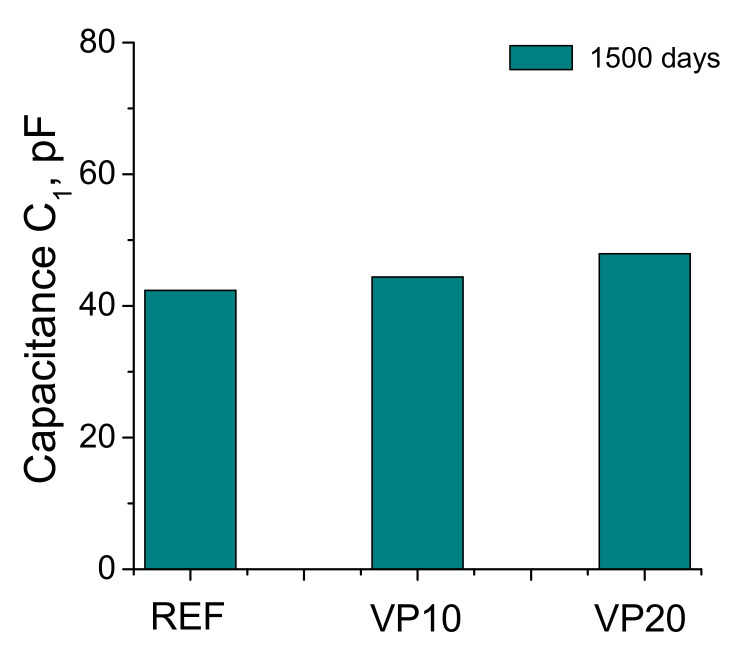
Results of capacitance C_1_ for the mortar series studied.

The results of capacitance C_1_ can be observed in Figure 6. This capacitance was slightly higher as the percentage of volcanic powder in the mortars rose. Nevertheless, very low differences between the different binders have been noted in the results of capacitance C_1_.

**Figure 7:**
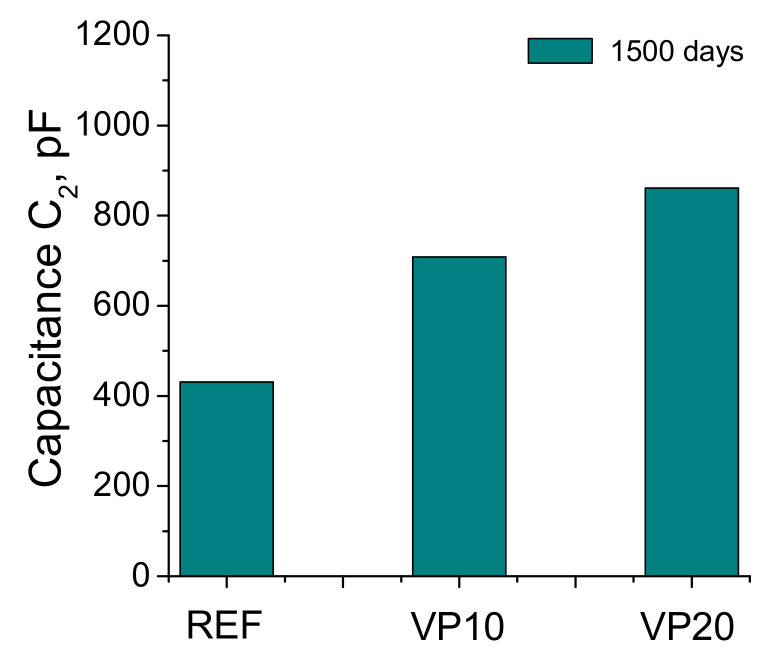
Capacitance C_2_ values for the REF, VP10 and VP20 specimens.

In relation to capacitance C_2_, its results have been represented in Figure 7. The greatest value of this capacitance was observed for the VP20 series, followed by VP10. The mortars of the REF series showed the lowest value of this parameter.

**Figure 8:**
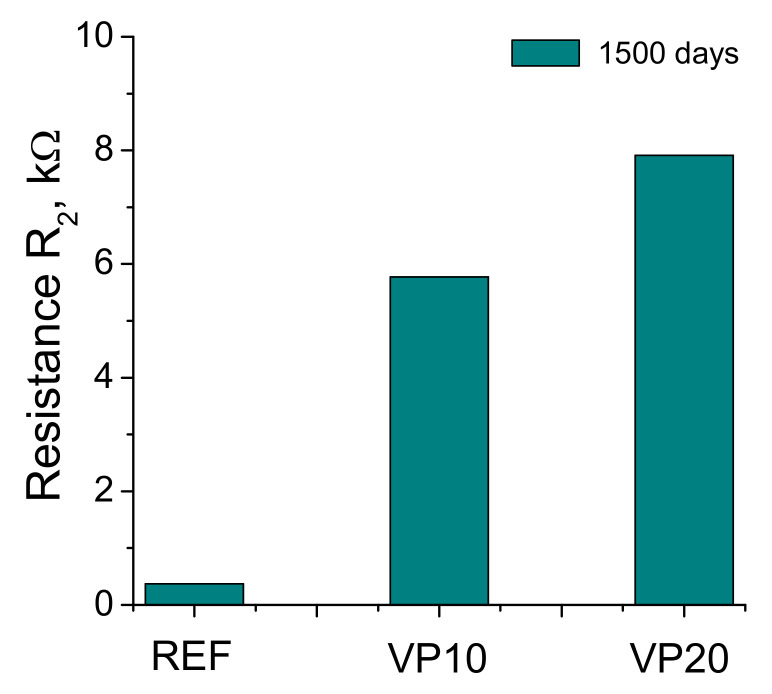
Resistance R_2_ results noted for the mortars tested.

The results of impedance resistance R_2_ can be observed in Figure 8. This resistance was higher for specimens with volcanic powder after 1500 hardening days, in comparison with reference ones. In addition to this, the value of this parameter increased with the volcanic powder proportion in the binder, showing the VP20 mortars the greatest resistance R_2_.

### 3.3. Differential Thermal Analysis

**Figure 9:**
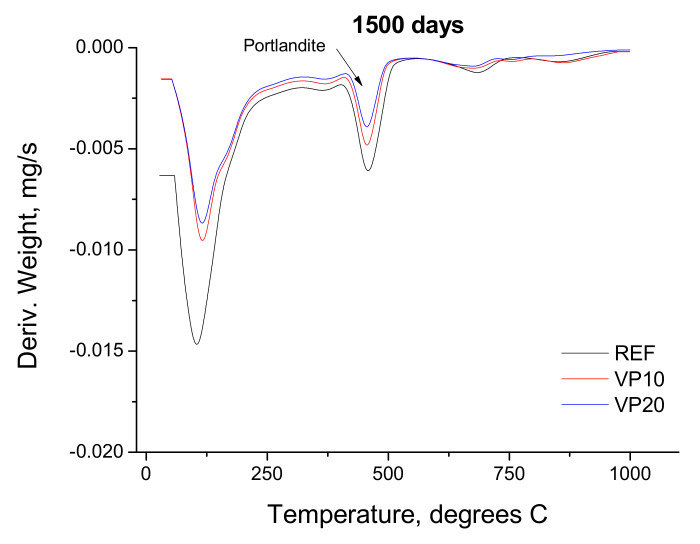
Derivate of weight versus temperature curve obtained for REF, VP10 and VP20 mortars after 1500 hardening days.

The derivate of the weight versus temperature curves obtained for the analyzed mortar series after 1500 days are depicted in Figure 9. The portlandite peak area of this curve was lower as the percentage of clinker replacement by volcanic powder increased.

### 3.4. Steady-State Chloride Diffusion Coefficient

**Figure 10:**
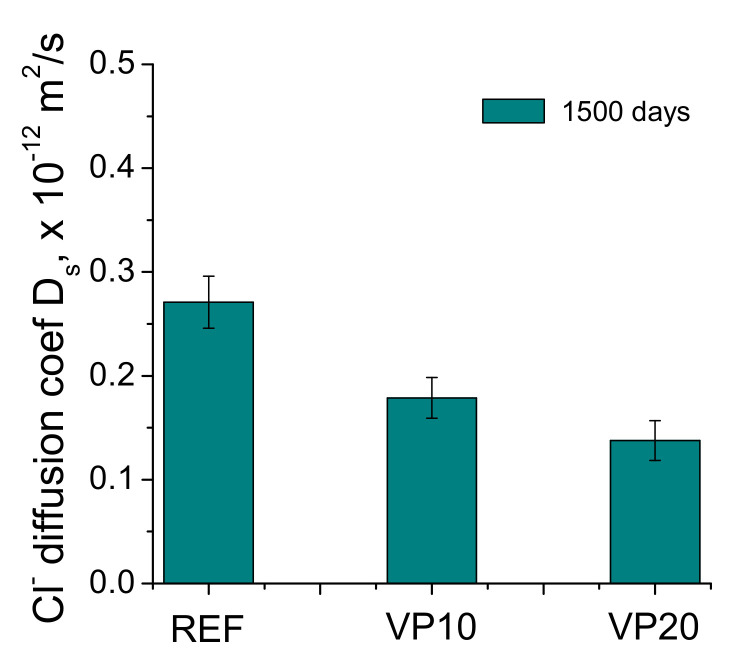
Steady-state chloride diffusion coefficients noted for the binders tested.

The results of the steady-state chloride diffusion coefficient obtained from the sample’s resistivity for the analyzed series in the very long term have been represented in Figure 10. This coefficient was greater for mortars taken as a reference, in comparison to those which incorporated volcanic powder. The lowest diffusion coefficient was observed for specimens with the greater percentage of volcanic powder (VP20 mortars).

### 3.5. Water Absorption after Immersion

**Figure 11:**
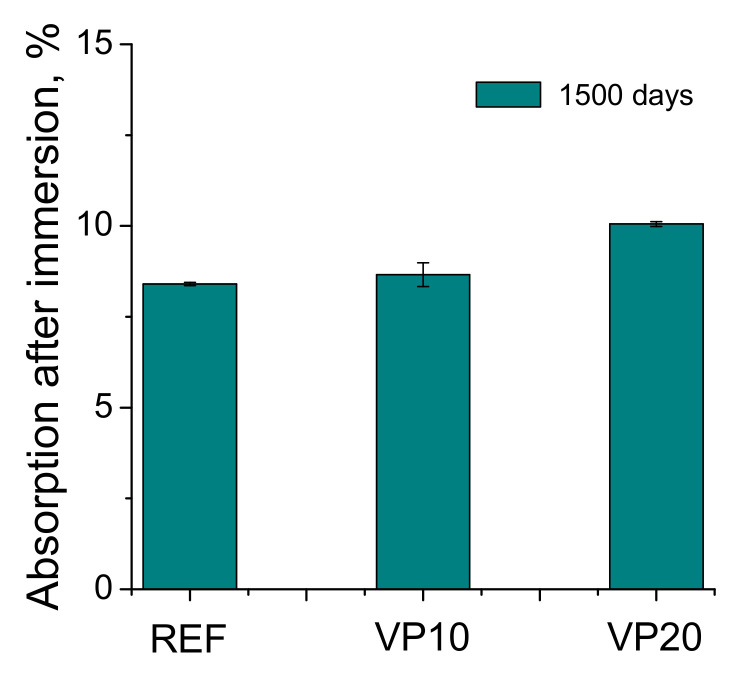
Percentage of absorption after immersion for the series analyzed.

The percentages of water absorption after immersion, obtained for the mortar series analyzed after 1500 hardening days, are represented in Figure 11. This absorption was slightly greater for VP20 series with respect to REF and VP10 mortars. In spite of this, the differences between the three binders studied were overall scarce.

### 3.6. Shrinkage/Expansion

**Figure 12:**
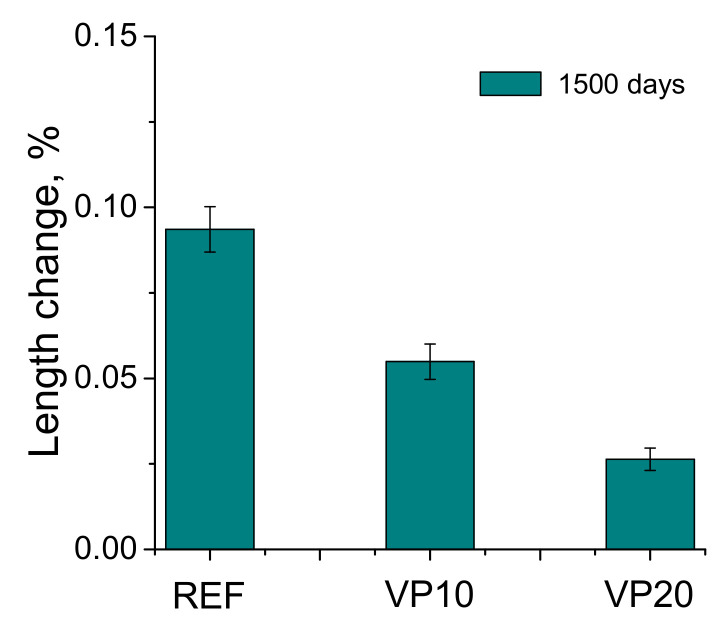
Length change noted for the mortar series analyzed after 1500 hardening days.

The percentage of length change noted for the different binders studied is depicted in Figure 12. All the mortars showed an expansion during the analyzed time period. The greatest expansion was obtained for the reference series, while the smallest corresponded to the VP20 binder.

## 4. Discussion

### 4.1. Microstructure Characterization

Regarding the mercury intrusion porosimetry technique, the similar total porosity in the very long term noted for the mortars tested (see Figure 3) would indicate that they would have a similar fraction of solids and overall volume of pores, independently of the binder used. In this regard, the incorporation of 20% of volcanic powder in the binder would not entail a noticeable increase in the porosity, compared to reference specimens, in keeping with other research [26]. On the other hand, the distributions of pores by size obtained with this technique, after 1500 hardening days (see Figure 4), would reveal a greater pore refinement in the microstructure produced by the volcanic powder addition, as would suggest the greater proportion of finer pores, mainly those with sizes lower than 10 nm, noted for the VP10 and VP20 specimens, in comparison with reference ones. In addition to this, the results of the percentage of Hg at the end of the mercury intrusion porosimetry test (see Figure 5) would corroborate this higher pore refinement of mortars with volcanic powder. This parameter gives data regarding the tortuosity of the pore structure of the material [41]. Therefore, the highest percentages of Hg retained, obtained in the VP10 and VP20 samples, would indicate more tortuosity in their microstructure, directly related to their high pore refinement. The results of pore size distributions and Hg retained would also show that the microstructure refinement was greater as the proportion of clinker replaced by volcanic powder increased.

The microstructure refinement produced by the addition of volcanic powder after 1500 hardening days could be a result of the development of its pozzolanic reactions. These reactions would form solid phases as products, closing the pore network of the mortars and increasing the relative presence of pores with lower diameters. This pozzolanic activity of the volcanic powder after 4 hardening years would also be suggested by the differential thermal analyses performed. With this technique, it has been noted lower areas of the portlandite peak in the curves derivate of the weight versus the temperature for series with volcanic powder, compared to those without an addition (see Figure 9). This pozzolanic activity would be in keeping with other research [20,21], in which the effects of additions coming from volcanic materials in the short term have been analyzed.

Furthermore, the results of impedance spectroscopy showed coincidences with the mercury intrusion porosimetry results previously discussed. On one hand, the parameter C_1_ gives data regarding global fraction of solids in the sample [41], independently of its pore size distribution. As has been previously described, scarce differences between the different studied mortars were observed in this capacitance (see Figure 6). This would suggest that global fractions of solids were very similar after 1500 hardening days for the different studied series. This result would agree with that obtained for porosity, which also revealed slight differences in the total volume of pores, independently of the addition of volcanic powder. The incorporation of volcanic powder produced scarce effects on the global porosity, whereas it increased the refinement of the microstructure, as noted by other supplementary cementitious materials, such as fly ash [50].

The impedance capacitance C_2_ is associated with the internal pores surface contacting with the electrolyte, which fills the material’s pore network [42]. Therefore, a rise in this surface produced by the formation of solids as rough structures on the preexisting walls of the pores, due to the progressive development of pozzolanic and hydration reactions, would entail higher values of this capacitance C_2_ [43]. Furthermore, for specimens with similar global porosity and greater pore refinement, the higher presence of finer pores would also produce a higher global pore surface, giving greater capacitance to C_2_ values. This parameter showed higher values for VP10 and VP20 specimens after 1500 hardening days compared to reference mortars, which became greater as the percentage of volcanic powder in the binder rose (see Figure 7). This result would indicate that the internal pore surface in specimens with this addition was higher than that for referenced ones, suggesting that the addition of volcanic powder would produce a greater microstructure refinement, as a consequence of the progress of its pozzolanic reactions [20,21]. This would agree with the pore size distributions and Hg retained results previously discussed.

Finally, the R_2_ resistance gives information about the material’s pore structure [42], The values of R_2_ are higher as greater is the presence of pores with smaller diameters. As previously described, the specimens with volcanic powder showed higher resistances R_2_ in comparison with the reference ones (see Figure 8), and the highest values of this resistance corresponded to VP20 mortars. These results would also coincide with those discussed for capacitance C_2_, pore size distributions and percentage of Hg retained, revealing the pore refinement produced by this addition.

### 4.2. Durability-Related Parameters

With regard to the analyzed durability-related parameters of the series with volcanic powder in the very long term, the steady-state chloride diffusion coefficient exhibited smaller values for VP10 and VP20 specimens, in comparison with referenced ones (see Figure 10). As happened with some microstructural parameters, this coefficient decreased as the content of volcanic powder in the mortars increased. This result would agree with those obtained by other authors at short hardening ages [22,23]. It may be related to the high microstructural refinement observed for VP10 and VP20 specimens, which could be a consequence of the development of the volcanic-powder pozzolanic reactions [20,21] already discussed. The presence of a noticeable percentage of pores with lower sizes would entail a more difficult diffusion of aggressive chloride ions throughout the material, which would lead to lower values of the steady-state diffusion coefficient, as previously observed. This result would be relevant, because chlorides constitute one of the most damaging agents which could affect the durability of cementitious materials and, as was observed, the addition of volcanic powder as clinker replacement, improved the performance of the mortars at this point after1500 hardening days.

In relation to the percentage of absorption after immersion, in general, the differences between the studied mortars were scarce (see Figure 11), which would be in keeping with the results of impedance capacitance C_1_, as well as total porosity, suggesting that the overall volume of pores in the studied series was relatively similar, irrespective of the binder. In view of the length change results observed for the different series studied (see Figure 12), a length increase was noted for all of them, showing a slight expansion after 1500 hardening days. This could be expected due to the storage of the specimens at an optimum condition during the studied time period. Despite that, it is noticeable that mortars with volcanic powder showed less deformation than referenced ones.

Lastly, considering the results previously discussed, the incorporation of volcanic powder from the Calbuco volcano in mortars, with up to 20% of clinker replacement, would have beneficial effects after 1500 hardening days, in terms of the refinement of pore network and chloride diffusion. Furthermore, this addition would not noticeably worsen other properties, such as total porosity and water absorption by immersion. It is important to highlight that these results, in the very long term, would be interesting for evaluating the possible application of volcanic powder coming from the Calbuco volcano in real cement-based construction elements, which are generally required over a long service life. Furthermore, the use of this volcanic powder as a clinker replacement would also contribute to sustainability, providing additional advantages in relation to the protection of the environment.

## 5. Conclusions

The main conclusions that can be obtained from the results previously discussed can be summed up as follows:

- Mortars with the addition of volcanic powder from the Calbuco volcano showed a more refined pore network after 1500 hardening days, in comparison with referenced specimens, in view of the results of the impedance C_2_ capacitance and R_2_ resistance, as well as the pore size distributions obtained by mercury intrusion porosimetry;
- The higher microstructure refinement produced by the incorporation of volcanic powder from the Calbuco volcano could be due to the pozzolanic activity of this addition, as indicated by the differential thermal analyses after 1500 hardening days, which produced a greater proportion of finer pores;
- The global porosity and solid fraction was very similar for all of the studied mortars after 1500 days, irrespective of the presence of volcanic powder in the binder, as showed in the total porosity results, obtained from mercury porosimetry, and the impedance capacitance C_1_;
- The use of binders with volcanic powder from the Calbuco volcano produced a reduction of the steady-state chloride diffusion coefficient after 4 years, compared to mortars without this addition. Moreover, this parameter was lower as the percentage of volcanic powder in the binder rose. This improvement in the behaviour regarding the chloride diffusion could be produced by the more refined pore network caused by the addition of volcanic powder in the mortars;
- The incorporation of volcanic powder did not noticeably affect the performance of the mortars with respect to the water absorption after immersion in the very long term, suggesting an overall volume of pores that is fairly similar for the analyzed series;
- In accordance with the results obtained in this work, the 10% and 20% clinker replacement by volcanic powder from the Calbuco volcano would have beneficial effects in the performance of mortars after approximately 4 hardening years, especially regarding their microstructure development and chloride ingress resistance, without considerably affecting their water absorption, with the additional advantages regarding the protection of the environment.

## Figures and Tables

**Table 1:** Chemical components of volcanic powder.

Components	Value
Fe_2_O_3_	11.00%
CaO	8.27%
Al_2_O_3_	14.54%
SiO_2_	57.76%
K_2_O	2.14%
Na_2_O	2.41%
MgO	2.44%
TiO_2_	1.42%

## Data Availability

Data sharing not applicable.
